# Reciprocal change of occipitocervical parameters after anterior cervical discectomy and fusion

**DOI:** 10.1038/s41598-021-85189-3

**Published:** 2021-03-11

**Authors:** Eugene J. Park, Seungho Chung, Woo-Kie Min

**Affiliations:** Department of Orthopedic Surgery, Kyungpook National University Hospital, Kyungpook National University School of Medicine, (41944) 130 Dongdeok-ro, Jung-gu, Daegu, Republic of Korea

**Keywords:** Anatomy, Medical research

## Abstract

To evaluate the reciprocal changes in occipitocervical parameters according to the recovery of cervical lordosis (CL) after anterior cervical discectomy and fusion (ACDF) in patients with sagittal imbalance. Sixty-five cases that underwent ACDF were followed. They were divided according to the recovery of the CL: Group 1 (ΔCL > 5°, 30 cases) and Group 2 (ΔCL < 5°, 35 cases). The following parameters were measured: occiput-cervical inclination (OCI), CL, occiput-C2 angle (OC2A), distance between external occipital protuberance and spinous process of C2 (OC2D), distance between spinous processes of C2 and C7 (C27D), and shortest distance between the plumb line of C2 body and posterosuperior corner of C7 (C27SVA). Overall, all parameters changed significantly after ACDF. Preoperative CL and preoperative C27D showed a correlation with ΔCL. ΔCL was negatively correlated with ΔC27D and ΔC27SVA. In Group 1, CL increased from − 2.60 ± 1.88° to 11.57 ± 1.83°, OC2A decreased from 23.96 ± 2.05° to 19.87 ± 1.36°, OC2D increased from 82.96 ± 1.48 mm to 86.50 ± 1.81 mm, C27D decreased from 95.61 ± 2.66 mm to 87.01 ± 2.50 mm, and C27SVA decreased from 24.14 ± 2.20 mm to 17.06 ± 2.14 mm. In Group 2, only OCI decreased significantly after ACDF. ACDF can increase CL postoperatively in patients with cervical sagittal imbalance. Patients with significant CL recovery after ACDF showed a reciprocal change in occipitocervical parameters. (OC2A, OC2D).

## Introduction

Nowadays, the importance of cervical spinal balance in sagittal plane is emphasized, and many studies have been reported^1–7^. The global spinal sagittal balance is generally explained by the relationship between the plumb line from the second vertebral body and first sacral body^8^. It is reported that the change in cervical lordosis (CL) may lead to a reciprocal change in the global sagittal alignment^9^. Loss of CL due to degenerative changes may advance with aging, which may deteriorate sagittal balance, and result in excessive fatigue in muscle groups supporting the occiput and posterior neck and can cause chronic neck pain^6^. CL is considered one of the essential cervical sagittal parameters and vital for long-term outcomes. Although CL is an essential parameter, unlike the nomenclature, the majority of the healthy population does not have lordotic curvature in the cervical spine^5,10^. Additionally, rather than CL, quality of life (QOL) was comprehensively correlated with sagittal occiput-C2 angle (OC2A) and C2–7 sagittal vertical axis (C27SVA)^4^. In other studies, the amount of CL was not correlated to C27SVA but was considered a consequence of SVA and T1 slope (T1S)^5,8,11^. However, due to its feasibility, CL is still considered one of the commonly used cervical sagittal parameters^1–3^.

Anterior cervical discectomy and fusion (ACDF) is the most common spinal surgery for the treatment of cervical degenerative disease. ACDF decompresses the spinal canal and foramina through removal of the intervertebral disc with body spurs and induces segmental fusion by grafting in the intervertebral space. It restores the disc height and cervical sagittal alignment, especially CL^12,13^ Meanwhile, horizontal gaze is one of the functions of the cervical spine and related to QOL^1,4^. The cervical spine is known to change its curvature in order to maintain a horizontal gaze. Recent study showed changes to subaxial cervical curvature and occipitocervical segment is known to be negatively correlated to each other^3^. Considering that ACDF can restore CL^13,14^, our hypothesis was that ACDF would improve CL postoperatively and subsequently negatively influence the upper cervical sagittal alignment. This study was aimed to evaluate the change in radiographic cervical sagittal parameters using plain lateral radiographs of patients undergoing ACDF.

## Material and methods

This study was a retrospective electronic medical chart review study. We performed this study according to the Declaration of Helsinki. Approval of the Institutional Review Board of Kyungpook National University Hospital was obtained before performing this study. The need for informed consent was waived due to the retrospective nature of the study by the institutional ethics committee (institutional review board of Kyungpook National University Hospital) (IRB File No. 2020-05-028).

### Patient selection

By setting the effect size as 0.5, alpha error probability as 0.05, and power (1-beta error probability) as 0.8 using G*Power program running t-tests for matched pairs, we obtained a result that at least 27 samples were necessary for this study. At least 30 heterogeneous samples with three raters were required for the analysis of the reliability study^15^. We retrospectively reviewed 135 patients that underwent ACDF using cage and plate from November 2004 to April 2018. The inclusion criteria were patients with at least 1-year postoperative follow-up plain lateral cervical X-ray with preoperative sagittal imbalance in terms of C27SVA. C27SVA is the shortest distance between the C2 plumb line and posterosuperior corner of the C7 body. A positive value indicates C2 plumb line being ventral to the posterosuperior corner of the C7 body, and a negative value indicates vice versa. The definition of sagittal imbalance was C27SVA > 15 mm. The exclusion criteria were surgery at three or more levels, fracture, infection, additional posterior surgery, and lack of visualization of the index cervical parameters on the lateral plain radiograph.

### Operative technique

The patient was placed in supine position with padding beneath the posterior neck. The index level was identified on the lateral view of the fluoroscope. Transverse skin incision along the skin crease was made. After longitudinal platysma splitting, a standard Smith-Robinson approach was performed. Discectomy and endplate cartilage removal were performed. In patients with cervical spondylotic radiculopathy or amyotrophy, longus colli muscles were dissected more laterally until full exposure of the uncovertebral joint to perform additional uncinectomy. Polyetheretherketone (PEEK) cage (Cervios ChronOS, Synthes GmbH, Oberdorf, Switzerland), allogenic fibular bone (Matrispine, Lifenet Health, Virginia Beach, VA, USA), or autogenous iliac strut bone was used for an interbody spacer. PEEK cage had 7.5° of lordosis, allogenic fibular bone had 7° of lordosis, and autogenous iliac strut bones were trimmed to the desired angle of the index disc space. To minimize adjacent segment disease, the height of the plate was adjusted that the distance between the plate and adjacent disc space was at least 5 mm on the sagittal plane. Patients were recommended to use the Philadelphia brace for 6 weeks after surgery.

### Radiographic evaluation

**Figure 1:**
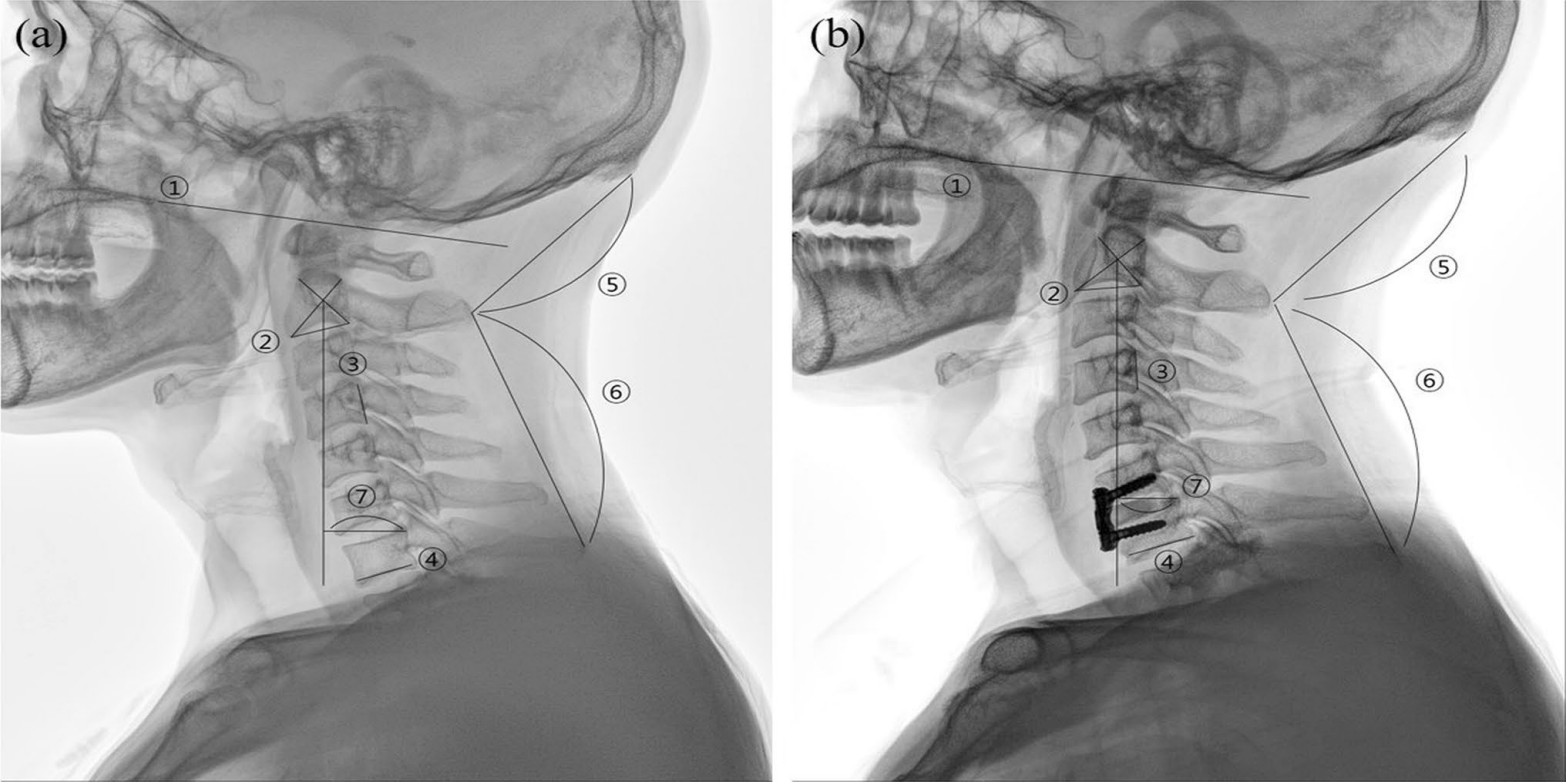
Example of measurements and landmarks of the occipitocervical and subaxial cervical parameters. A 55-year-old man who underwent ACDF on C6-7 due to soft disc herniation (**a**) Preoperative lateral X-ray (**b**) Postoperative follow-up lateral X-ray. Note the increment of CL and OC2D and decrement of OC2A and C27SVA. ① McGregor line ② C2 lower endplate ③ posterior border of the fourth cervical vertebral body ④ C7 lower endplate ⑤ distance between external occipital protuberance and most prominent portion of C2 spinous process (OC2D) ⑥ distance between the C2 and C7 spinous process (C27D) ⑦ shortest distance between the plumb line from the center of second cervical vertebrae body and posterosuperior corner of seventh cervical vertebrae body (C27SVA). OCI, angle between ① and ③; OC2A, angle between ① and ②; CL, angle between ② and ④. Lordotic angle is measured as positive value, and kyphotic angle is measured as negative value. Positive C27SVA indicates that the C2 plumb line is anterior to posterosuperior corner of C7 body and vice versa. ACDF, anterior cervical discectomy and fusion; CL, cervical lordosis; OC2D, occiput-C2 distance; OC2A, occiput-C2 angle; C27SVA, C2–7 sagittal vertical axis; C27D, C2–C7 interspinous process distance; OCI, occipitocervical inclination.

All measurements were performed by a picture-archiving communication system (PACS) (INFINTT PACS; INFINITT Healthcare, Seoul, Korea). We collected the preoperative and postoperative follow-up plain radiographs of the lateral cervical spine. The patients were placed on a comfortable standing position while maintaining a horizontal gaze while the radiographs were obtained. Occipitocervical inclination (OCI), CL, OC2A, occiput-C2 interspinous process distance (OC2D), C2-7 interspinous process distance (C27D), and C27SVA were measured. The measuring methods for each parameter were as follows (Fig. 1):

1. OCI: The Cobb angle formed by the line parallel to the posterior cortical bone of the fourth cervical vertebral body and the line connecting the posterior end of the hard palate and the most caudal point of the occipital curve (McGregor line)^16^.
2. CL: The Cobb angle formed by the line parallel to the inferior endplate of the second cervical vertebral body and the line parallel to the lower or upper endplate of the seventh cervical vertebra. A negative value indicates a kyphotic angle.
3. OC2A: The Cobb angle formed by McGregor line, and the line parallel to the lower endplate of the second cervical vertebra.
4. OC2D: The distance from the external occipital protuberance to the most posterior prominent point of the C2 spinous process.
5. C27D: The distance between the most posterior prominent points of C2 and C7 spinous processes.
6. C27SVA: The shortest distance between the C2 plumb line from the center of the body and posterosuperior corner of the C7 body. A sagittal imbalance was defined as a value > 15 mm.

### Statistical analysis

Statistical analysis was performed using SPSS version 20.0 (IBM, Armonk, NY, USA). A paired t-test or Wilcoxon sign rank test was used for change in cervical parameters. Student’s t-test and independent t-test were performed for continuous variables, and chi-square test was performed for categorical variables. A *p*-value < 0.05 was considered statistically significant. The intraclass correlation coefficient was calculated based on absolute agreement and two-way mixed-effects model. Values < 0.5, between 0.5 and 0.75, between 0.75 and 0.9, and > 0.9 indicated poor, moderate, good, and excellent reliability, respectively^15^.

## Results

After selecting the patients according to our criteria, a total of 65 patients were eligible in this study. We divided the patients according to the recovery of CL postoperatively. Patients with postoperative CL change (ΔCL) of ≥ 5° were designated as Group 1, and patients with ΔCL < 5° were designated as Group 2. Positive value of ΔCL indicated postoperative increment and vice versa. The demographics and operative details of both groups are described in Table 1. All variables did not show a significant difference between the two groups.

**Table 1:** Comparison of demographic and surgical data of both groups.

	Group 1 (*n* = 30)	Group 2 (*n* = 35)	*p* value
Age (years)	51.1 ± 12.9	55.2 ± 12.1	0.195
**Gender**			
Male	18	23	0.634
Female	12	12	
**Number of level(s)**			
1	21	31	0.062
2	9	4	
**Involved level(s)**			
C23	1	0	0.227
C34	2	4	
C45	10	8	
C56	17	19	
C67	9	10	
C7T1	0	1	

All continuous values are in mean ± standard deviation. Involved level(s) include each level for multilevel surgery. *P* value below 0.05 is considered statistically significant. PEEK, polyetheretherketone.

### Overall

All six cervical sagittal parameters (OCI, CL, OC2A, OC2D, C27D, and C27SVA) changed significantly after surgery. OCI decreased from 107.07 ± 6.56° to 105.15 ± 6.25°, CL increased from 4.40 ± 12.40° to 10.64 ± 10.00°, OC2A decreased from 22.86 ± 8.95° to 20.46 ± 7.42°, OC2D increased from 82.48 ± 1.48 mm to 84.63 ± 1.30 mm, C27D decreased from 91.84 ± 14.06 mm to 88.43 ± 12.51 mm, and C27SVA decreased from 23.90 ± 9.40 mm to 20.08 ± 11.54 mm (Table 2).

**Table 2:** Overall patients change of cervical sagittal parameters.

	Preoperative (*n* = 65)	Postoperative (*n* = 65)	*p* value
OCI (°)	107.07 ± 6.56	105.15 ± 6.25	0.009
CL (°)	4.40 ± 12.40	10.64 ± 10.00	0.000
OC2A (°)	22.86 ± 8.95	20.46 ± 7.42	0.005
OC2D (mm)	82.48 ± 1.48	84.63 ± 1.30	0.001
C27D (mm)	91.84 ± 14.06	88.43 ± 12.51	0.002
C27SVA (mm)	23.90 ± 9.40	20.08 ± 11.54	0.003

All continuous values are in mean ± standard deviation. Values for OCI, CL, and OC2A are degrees, and for OC2D, C27D, and C27SVA are millimeters. OCI, occipitocervical inclination; CL, cervical lordosis; OC2A, occiput-C2 angle; OC2D, occiput-C2 spinous process distance; C27D, C2-C7 interspinous process distance; SVA, C2-C7 sagittal vertical axis. *p* value below 0.05 is considered statistically significant.

**Figure 2:**
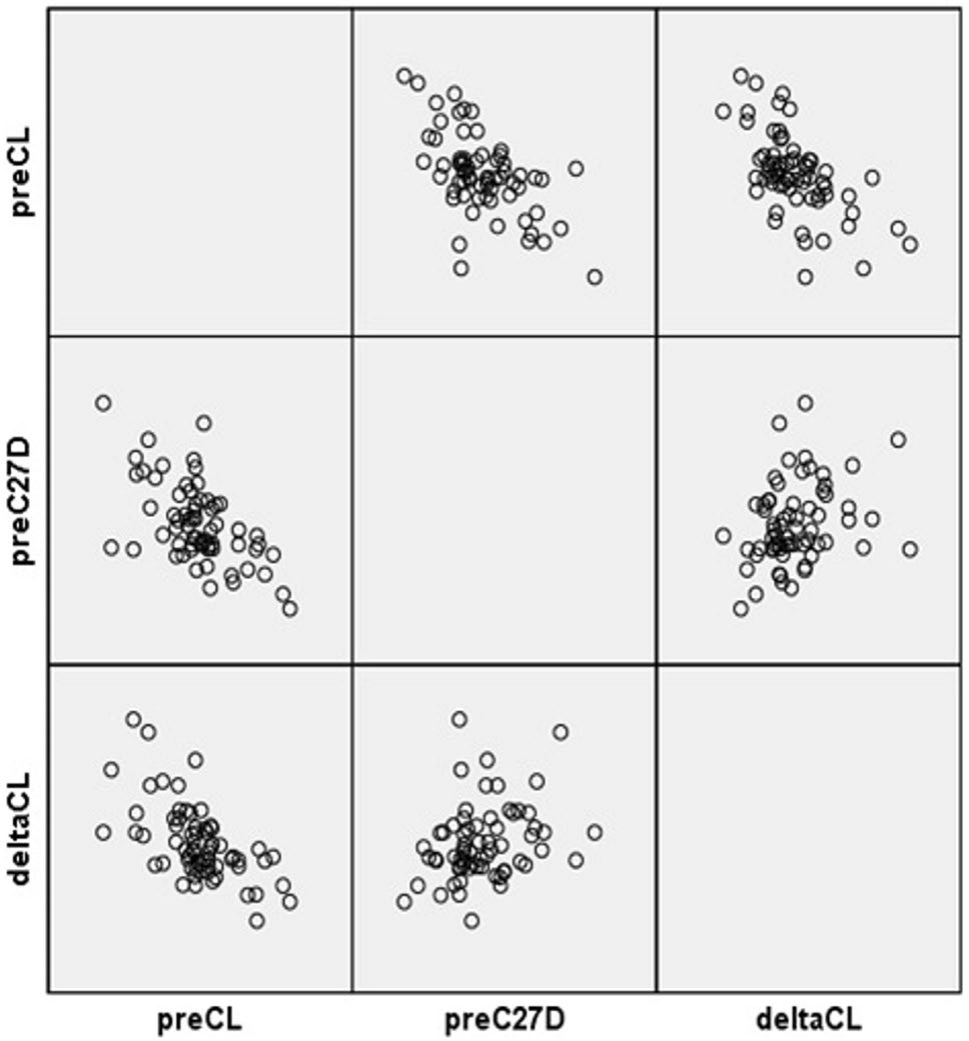
Scatterplot of preoperative CL and ΔCL. A significant negative correlation was found. Δ (delta), postoperative–preoperative value; CL, cervical lordosis.

**Figure 3:**
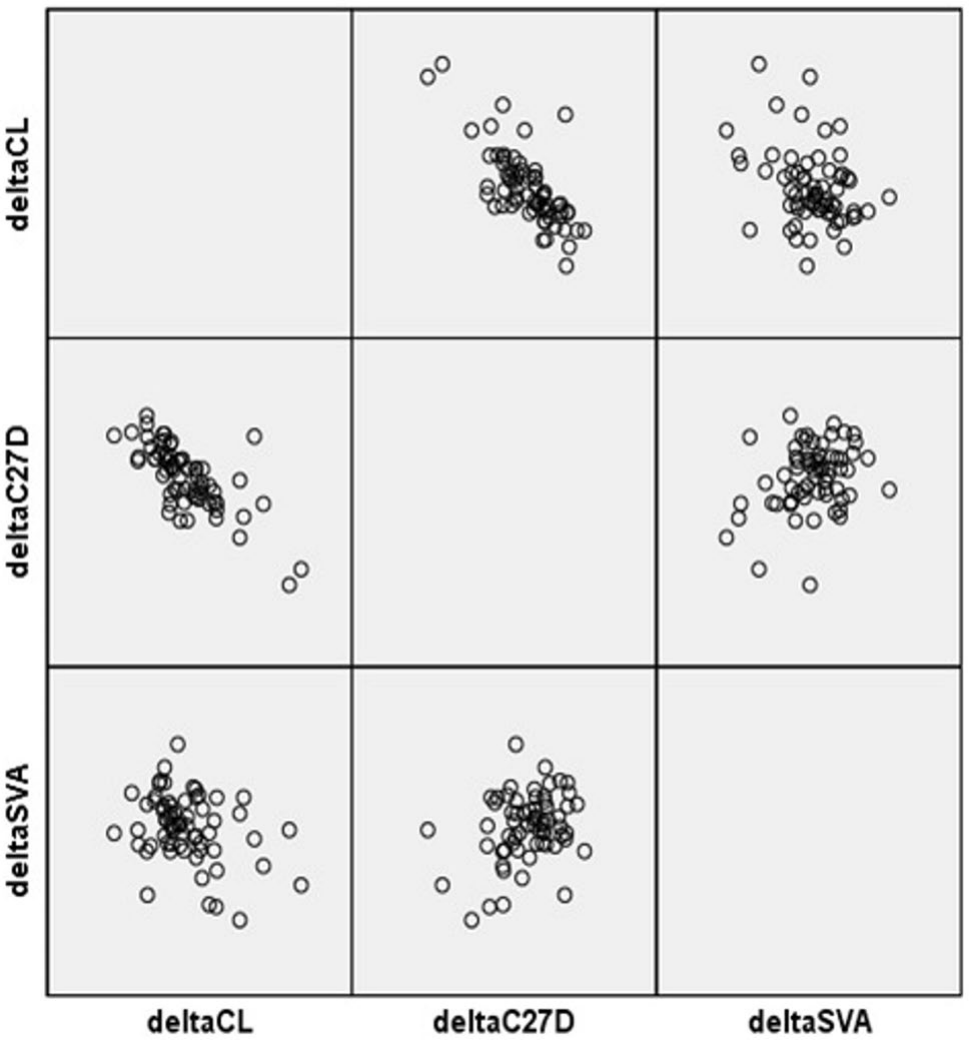
Scatterplot matrix of ΔCL, ΔC27D, and ΔC27SVA. A significantly negative correlation was found between ΔCL and both ΔC27D and ΔC27SVA. Δ (delta), postoperative–preoperative value; CL, cervical lordosis; C27D, C2–7 interspinous process distance; C27SVA, C2–7 sagittal vertical axis.

The overall correlation between preoperative and change in parameters are summarized in Table 3. Preoperative CL and preoperative C27D showed a correlation with ΔCL (r =  − 0.413 and p = 0.000, and r = 0.210 and p = 0.013, respectively) (Fig. 2). In other words, lower preoperative CL and higher preoperative C27D showed more increment of CL postoperatively. ΔCL was negatively correlated with ΔC27D and ΔC27SVA (r =  − 0.551, p = 0.000 and r =  − 0.206, p = 0.015, respectively) (Fig. 3). In other words, more increase of CL was related to more decrease in C27D and C27SVA. Preoperative CL was significantly lower and preoperative C27D was significantly higher in Group 1 compared to Group 2. Other preoperative radiographic parameters were not significantly different between the two groups (Table 4).

**Table 3:** Correlation between preoperative parameters and Δ values of each parameter.

	preOCI	preCL	preOC2A	preOC2D	preC27D	preSVA	ΔOCI	ΔCL	ΔOC2A	ΔOC2D	ΔC27D	ΔSVA
**preOCI**												
Corr.	1.000	0.072	0.370**	− 0.114	− 0.073	0.221**	− 0.286**	− 0.057	− 0.155	0.161	0.096	0.194*
Sign.		0.396	0.000	0.178	0.393	0.009	0.001	0.504	0.067	0.058	0.260	0.022
**preCL**												
Corr.		1.000	− 0.171*	0.010	− 0.362**	− 0.024	− 0.032	− 0.413**	0.075	− 0.064	0.301**	0.112
Sign.			0.044	0.910	0.000	0.781	0.709	0.000	0.380	0.451	0.000	0.189
**preOC2A**												
Corr.			1.000	− 0.261**	− 0.018	0.115	− 0.089	0.068	− 0.242**	0.224**	− 0.051	0.136
Sign.				0.002	0.834	0.176	0.292	0.421	0.004	0.008	0.545	0.110
**preOC2D**												
Corr.				1.000	0.204*	− 0.059	0.050	0.055	0.154	− 0.313	− 0.185	− 0.079
Sign.					0.017	0.486	0.556	0.519	0.069	0.000	0.029	0.353
**preC27D**												
Corr.					1.000	− 0.069	− 0.034	0.210*	− 0.028	− 0.033	− 0.339	− 0.018
Sign.						0.415	0.688	0.013	0.743	0.700	0.000	0.834
**preSVA**												
Corr.						1.000	− 0.076	0.044	− 0.044	− 0.034	0.010	− 0.156
Sign.							0.374	0.606	0.602	0.692	0.910	0.066
**ΔOCI**												
Corr.							1.000	0.046	0.343**	− 0.280**	0.034	0.013
Sign.								0.587	0.000	0.001	0.688	0.883
**ΔCL**												
Corr.								1.000	− 0.138	0.152	− 0.551**	− 0.206*
Sign.									0.104	0.075	0.000	0.015
**ΔOC2A**												
Corr.									1.000	− 0.413	0.138	0.104
Sign.										0.000	0.105	0.219
**ΔOC2D**												
Corr.										1.000	− 0.81	− 0.034
Sign.											0.342	0.688
**ΔC27D**												
Corr.											1.000	0.186*
Sign.												3028
**ΔSVA**												
Corr.												1.000
Sign.												

Pre, preoperative; Δ, postoperative value–preoperative value; Corr., correlation efficient, Sign., *p*-value. *: *p*-value < 0.05, **: *p*-value < 0.001.

**Table 4:** Preoperative values of cervical sagittal parameters of both groups.

	Group 1 (*n* = 30)	Group 2 (*n* = 35)	*p* value
OCI (°)	106.90 ± 6.14	107.21 ± 6.99	0.853
CL (°)	− 2.59 ± 1.88	10.39 ± 1.84	0.000
OC2A (°)	23.96 ± 2.05	21.92 ± 1.09	0.717
OC2D (mm)	82.96 ± 12.84	82.07 ± 11.35	0.768
C27D (mm)	95.61 ± 14.57	88.61 ± 12.94	0.045
C27SVA (mm)	24.14 ± 2.20	23.70 ± 1.10	0.590

All continuous values are in mean ± standard deviation. OCI, occipitocervical inclination; CL, cervical lordosis; OC2A, occiput-C2 angle; OC2D, occiput-C2 spinous process distance; C27D, C2-C7 interspinous process distance; SVA, C2-C7 sagittal vertical axis. *p* value below 0.05 indicates statistically significant difference between two groups.

### Stratification by ΔCL

#### Group 1 (ΔCL > 5°)

The change of CL, OC2A, OC2D, C27D, and C27SVA were statistically significant. (p = 0.000, p = 0.013, p = 0.005, p = 0.000, and p = 0.001, retrospectively). OCI decreased from 106.90 ± 1.12° to 104.98 ± 0.98°, CL increased from − 2.60 ± 1.88° to 11.57 ± 1.83°, OC2A decreased from 23.96 ± 2.05° to 19.87 ± 1.36°, OC2D increased from 82.96 ± 1.48 mm to 86.50 ± 1.81 mm, C27D decreased from 95.61 ± 2.66 mm to 87.01 ± 2.50 mm, and C27SVA decreased from 24.14 ± 2.20 mm to 17.06 ± 2.14 mm. However, the change in OCI was not significant (p = 0.137) (Table 5).

**Table 5:** Change of cervical sagittal parameters by group.

	Group 1 (*n* = 30)			Group 2 (*n* = 35)		
	Pre	Post	*p* value	Pre	Post	*p* value
OCI (°)	106.90 ± 6.14	104.98 ± 5.37	0.137	107.21 ± 6.99	105.29 ± 6.99	0.018
CL (°)	− 2.59 ± 1.88	11.57 ± 10.00	0.000	10.39 ± 1.84	9.85 ± 10.08	0.971
OC2A (°)	23.96 ± 2.05	19.87 ± 7.45	0.013	21.92 ± 1.09	20.96 ± 7.47	0.210
OC2D (mm)	82.96 ± 12.84	86.50 ± 9.93	0.005	82.07 ± 11.35	83.03 ± 10.88	0.079
C27D (mm)	95.61 ± 14.57	87.01 ± 13.71	0.000	88.61 ± 12.94	89.65 ± 11.44	0.342
C27SVA (mm)	24.14 ± 2.20	17.06 ± 11.72	0.001	23.70 ± 1.10	22.67 ± 10.88	0.482

All values are in mean ± standard deviation. OCI, occipitocervical inclination; CL, cervical lordosis; OC2A, occiput-C2 angle; OC2D, occiput-C2 spinous process distance; C27D, C2-C7 interspinous process distance; C27SVA, C2-C7 sagittal vertical axis. *p* value below 0.05 indicates statistically significant difference between preoperative and postoperative values.

#### Group 2 (ΔCL < 5°)

OCI decreased from 107.21 ± 1.18° to 105.29 ± 1.18°, CL decreased from 10.399 ± 1.84° to 9.85 ± 1.70°, OC2A decreased from 21.92 ± 1.09° to 20.96 ± 1.26°, OC2D increased from 82.07 ± 1.92 mm to 83.03 ± 1.84 mm, C27D increased from 88.61 ± 2.19 mm to 89.65 ± 1.93 mm, and C27SVA decreased from 23.70 ± 1.10 mm to 22.67 ± 1.84 mm. The change in CL, OC2A, OC2D, C27D, and C27SVA was not significant (p = 0.971, p = 0.210, p = 0.079, p = 0.342, and p = 0.482, respectively). Only, the change in OCI showed statistically significant change (p = 0.018) (Table 5).

### Intraclass correlation coefficient

The interobserver and intraobserver reliability of each parameter using preoperative radiographs are as follows. The interobserver reliabilities of preoperative OCI, OC2A, CL, OC2D, C27D, and C27SVA were 0.902, 0.981, 0.954, 0.879, 0.966, and 0.996, respectively, all showing excellent reliability. The intraobserver reliability of preoperative OCI, OC2A, CL, OC2D, C27D, and C27SVA by the board-certified orthopedic surgeon was 0.985, 0.981, 0.991, 0.857, 0.994, and 0.971, respectively, all showing excellent reliability except OC2D with good reliability.

## Discussion

Our study showed that ACDF increased CL and OC2D and decreased OCI, OC2A, C27D, and C27SVA postoperatively. Specifically, patients with ΔCL > 5° showed a significant change in all parameters except for OCI. On the other hand, patients with ΔCL < 5° showed no significant change in cervical sagittal parameters after surgery except for OCI.

Essentially, to maintain a horizontal gaze, the curvature of the cervical spine changes^17^. The classical parameter used for measurement of horizontal gaze is the chin-brow vertical angle (CBVA), with others including McGregor slope (McGS) and slope of the line of sight^4,18^. However, the landmarks of CBVA were not all readily measured on our plain radiographs. A study revealed that McGS was significantly correlated with CBVA; thus, McGS was measured in our study to evaluate the horizontal gaze^18^. Hasegawa et al. used EOS system in asymptomatic population to measure sagittal parameters, showing the mean value of McGS as 3.8°^10^. The measurements of preoperative and postoperative McGS in our study were 4.56 and 5.45°, respectively, which are similar to those measured in the asymptomatic population.

There are three main methods of measuring the CL: Cobb’s method, Harrison posterior tangent method, and Jackson’s physiological stress line method. Cobb’s method of CL can be measured at C1–7 or C2–7. CL using Cobb’s method in C1–7 tend to overestimate the angle, and that in C2–7 underestimates the angle, while the Harrison posterior tangent method provides the most accurate value. However, due to the advantages of picture archiving and communicating systems with good intra- and interobserver reliability, Cobb’s angle method is the most common measurement method^1,10^. Preoperative CL was negatively correlated with ΔCL, while preoperative C27D positively correlated with ΔCL. The flexion posture of the head increases the lower cervical foraminal areas^6^. Since the patients in this study had neurologic symptoms, preoperatively, patients tend to maintain flexion posture resulting in lower preoperative CL and higher C27D. We assume that, postoperatively, patients tend to recover CL not only because of the lordotic-shaped implants but also due to decompression of the neural elements.

Bao et al. divided individuals with or without neck symptoms and compared the sagittal parameters using biplanar stereoradiographic imaging. Among the numerous values, C27SVA, SLS, McGS, and TK showed a significant difference between the two groups, while CL did not. Especially, C27SVA was an independent predictor of cervical disability^4^. Miyazaki et al. analyzed the postoperative change in cervical alignments after cervical laminoplasty. They have concluded that the change in C27SVA was correlated with the change in OC2A. This reciprocal change was assumed to be attributed by maintaining the horizontal gaze of the individuals^17^. An experimental model of the cervical spine showed similar results. The increase in the C27SVA beyond the normal range subsequently increased OC2A^3^. Whereas cervical laminoplasty deteriorates C27SVA^19^, our results of ACDF show that C27SVA improves after surgery along with CL, which can be related to improved QOL.

The suboccipital muscles are a group of four muscles, located below the occipital bone, and involved in the extension and rotation of the occipitocervical junction. These include the rectus capitis posterior major, rectus capitis posterior minor, obliquus capitis superior, and obliquus capitis inferior. To assess the overall length of suboccipital muscles, we have measured the OC2D. Spinalis cervicis originate from the spinous process of C7 and attach to the spinous process of the axis, forming the lower part of the ligamentum nuchae. Splenius cervicis, trapezius, and semispinalis cervicis are also located in the posterior neck. We have measured the distance between the prominent posterior points of C2 and C7 spinous processes to distinguish the subaxial cervical movement from the occipitocervical junction. Flexion posture of the head may cause prolonged abnormal contraction of the suboccipital muscles^20^. In addition, the greater occipital nerve that resides in the suboccipital triangle may be impinged due to muscle contraction^6^. Our result showed increased OC2D and decreased C27D. We assume that increased OC2D will result in the relaxation of suboccipital muscles, which may, in turn, decompress the impinged greater occipital nerve. Decrement in C27D is considered a result of the improvement of CL and C27SVA, and such changes may lead to clinically favorable results.

OCI was introduced in order to account for the measurement of the occiptocervical alignment, which can be adopted during upper cervical fusion surgery^16^. It is the angle formed by the line connecting the posterior border of the C4 vertebral body and McGregor’s line. Measurement of OCI is easier to measure compared to the posterior occipitocervical angle (POCA)^21^, and can be measured consistently compared to occipitocervical distance (OCD), which is influenced by the morphologic variation of the C2 spinous process. OCI changes statistically significantly at the flexion and extension of the neck, and especially at the neutral position, is known to reflect CL. However, in our study, OCI did not change postoperatively in patients with significant recovery of CL, whereas it changed in patients without recovery of CL. The ramification of such a phenomenon is unclear.

There are a few limitations in our study. First, we could not involve the parameters, such as T1 slope and thoracic inlet angle. About half of patients with lateral radiographs were unable to measure such parameters. Since such values are correlated with cervical parameters^22^, future studies with larger series should be performed in the future. Second, we did not account for the correlation with spinopelvic parameters in this study. Pelvic incidence and tilt are reported to be correlated with CL and should be considered in a future study on cervical parameter changes^10,22^. Third, since this was a radiographic study, consideration of clinical outcomes should be analyzed in future studies. Finally, equivocal landmarks and heterogeneous morphology of landmarks made the measurement difficult. To minimize the errors, the observers placed preoperative and postoperative images simultaneously on the same monitor and zoomed in the images for precise and identical identification of the landmarks. OC2D showed lower reliability compared to other parameters, probably due to the equivocal reference point. Still, the inter- and intraobserver correlations were 0.879 and 0.857, respectively, showing “good” reliability. Whereas there was a study on reciprocal change of upper cervical alignment after posterior cervical surgery^17^, no previous study focused on such postoperative change after ACDF. The additional strength of our study is that we have adopted novel parameters using the distance between the landmarks of the occipitocervical and subaxial cervical region.

## Conclusion

ACDF can increase CL postoperatively in patients with cervical sagittal imbalance. Lower preoperative CL and higher preoperative C27D tended to show higher recovery of CL postoperatively. Patients with significant CL recovery after ACDF (> 5°) showed a reciprocal change in occipitocervical parameters (OC2A, OC2D).
